# The Effect of Different Compatibilizers on the Properties of a Post-Industrial PC/PET Blend

**DOI:** 10.3390/ma12010049

**Published:** 2018-12-24

**Authors:** Eleonora Dal Lago, Carlo Boaretti, Francesca Piovesan, Martina Roso, Alessandra Lorenzetti, Michele Modesti

**Affiliations:** Department of Industrial Engineering, University of Padova, Via Marzolo, 9-35131 Padova, Italy; eleonora.dallago@phd.unipd.it (E.D.L.); carlo.boaretti@unipd.it (C.B.); francesca.piovesan@unipd.it (F.P.); martina.roso@unipd.it (M.R.); alessandra.lorenzetti@unipd.it (A.L.)

**Keywords:** polycarbonate, polyethylene terephthalate, blend, mechanical recycling, chain extender, impact strength modifier

## Abstract

The substitution of virgin resins by recycled ones is a worldwide tendency that is supported by the fluctuation of oil prices and the transition to a circular economy. Polymeric blends have been intensively studied because of their ability to provide tailored properties for particular applications. However, in their design phases, the issue of end-life re-use had not been well addressed, and now difficulties in their recycling are arising. In this study, we investigated the effect of three different compatibilizers: two chain extenders (CEs), (1) a styrene-acrylic oligomer (ESAo), and (2) methylene diphenyl diisocyanate (MDI) and an impact strength modifier, (3) an ethylene copolymer (EMAco), for the recycle of a post-industrial polycarbonate/polyethylene terephthalate (PC/PET) blend. The materials were prepared by reactive extrusion and characterized by intrinsic viscosity (IV) measurements, mechanical tests, differential scanning calorimetry (DSC), Fourier transform infrared spectroscopy analysis (FTIR), and transmission electron microscopy (TEM). The introduction of each additive has been demonstrated to improve the compatibility between PET and PC in the post-industrial blend, leading to enhanced mechanical properties. The IV measurements increased to values that were comparable to the virgin material. In addition, CEs affected the crystallization of PET (as they reduced the degree of crystallinity), while EMAco acted as a nucleating agent. Morphological analysis enabled confirming the compatibilization effects induced by the tested additives.

## 1. Introduction

Polycarbonate (PC) and polyethylene terephthalate (PET) are thermoplastic polymers that are widely used in several application fields nowadays. PC is an amorphous engineering plastic employed in optical data storage devices, such as CDs or DVDs, and in many other industrial sectors, such as automotive, transportation, and building. PET is a semi-crystalline polyester whose applications mostly involves fibers, films, and bottles. Recently, the industrial interest in the PC/PET blends has grown up due to their overall balance of properties, which are capable of obtaining high-performance polymers. These materials combine the excellent impact toughness, high mechanical and thermal properties, and good dimensional stability of PC with the excellent chemical resistance of PET. Polymer blending is in fact a more economical method for making new materials or compounds compared to direct polymer synthesis. For this reason, a lot of research studies have been focused on the study of the best blending method of these polymers [1,2,3,4,5], and many studies evaluated the reactions that occur between them [6,7].

On the other hand, the increasing use of plastics in various industrial sectors affects the increasing amount of waste generated, which, according to the principles of sustainable development, should be recycled and appropriately managed. The use of these polymeric materials, with targeted properties for special applications (in this specific case, in the automotive sector), is dynamically increasing, and the resulting wastes are only to a small extent managed. The disposal of those materials represents a serious environmental issue, and their direct recycling in the production processes is still limited due to the severe degradation that such polymers are exposed to during molding operations, and which do not allow the reuse of the blend after the common physical recycling procedures [8,9,10,11,12,13,14]. Additionally, the mechanical recycling of blends presents further complications due to the complexity of the morphological structures between the polymeric phases, which need special precautions to be restored.

The reactive extrusion with some adequately selected additives might be the solution to these problems for several reasons: it is less expensive than solid-state processing, it is easier to apply in existing normal extrusion systems, and it is more environmentally friendly compared to chemical recycling. Although the specific literature for the mechanical recycling of PC is not yet well established, there are some studies about the use of reactive extrusion with chain extenders (CEs) for PET recycling. Generally, chain extenders are multifunctional compounds with low molecular weight that act as bridges between the degraded chains of the polymer, restoring the molecular weight and mechanical properties. Some popular chain extenders are diepoxides [15,16], diisocyanates, dianhydrides [17], carbodiimides, and (bis)oxazolines [18]. 

The use of chain extenders has been adopted by some researchers to improve the compatibilization between PC and PET during melt blending [19,20,21,22]; however, their use in the recycling operations of a post-consumer blend has not been studied yet.

Generally, polymer blends are characterized by excellent mechanical performances, and from this point of view, in particular in the automotive industry, the impact resistance plays an important role. To improve the impact toughness in engineering blends, a common strategy is the incorporation of impact modifiers such as rubber or other elastomers. Typical impact modifiers include poly(methyl methacrylate)-grafted-butadiene-styrene rubber (MBS), acrylonitrile-butadiene-styrene copolymer (ABS) with high polybutadiene content, ASA rubber (acrylate rubber), and styrene–ethylene/butylene–styrene triblock copolymer (SEBS). The phase separation between the polymers and the rubber is an important condition, and the mechanical resistance increases if the rubber has good adhesion to the matrix, optimized average particle size distribution, and a separating distance between the elastomeric zones. Also, in this case, the literature on the use of an impact-strength modifier to restore the properties of a post-consumer blend is absent.

In this study, three different compatibilizers were selected to restore the properties of a post-industrial PC/PET blend by reactive extrusion: an epoxy styrene-acrylic oligomer (ESAo), pure methylene diphenyl diisocyanate (MDI), and an ethylene methyl acrylate copolymer (EMAco). The first two were chosen because of their well-established effects in the chain-extending process of PET, while the third is an impact strength modifier. In this work, the influence of these additives was analyzed by rheological, mechanical, thermal, and morphological measurements to evaluate the effectiveness of the recycling process.

## 2. Materials and Methods 

### 2.1. Materials

The virgin material is Xenoy XL1339 (Sabic, Riyadh, Saudi Arabia) that is an unreinforced PC/PET blend, offering excellent impact performance over a wide temperature range. The industrial operations to whom such material is exposed are mainly injection molding, coating, and chromium plating. The material that is used in this work came from a collection of post-industrial (POST-I) wastes, of the same PC/PET blend, which were used in the automotive industry. The compatibilizers that were involved were Joncryl-ADR-4468 (ESAo) supplied by BASF (Ludwigshafen, Germany), which is an epoxy functional oligomeric acrylic with the following physical characteristics: M_w_ = 7250 Da, high number average functionality (ƒ_n_ > 4), EEW (epoxy equivalent weight) = 310 g/mol, and T_g_ = 59 °C, obtained in flake form; pure methylene diphenyl diisocyanate (MDI) from Sigma-Aldrich (St. Louis, MO, USA) with ƒ_n_ = 2 and Elvaloy AC 12024 (EMAco) supplied by DuPont (Wilmington, DE, USA), which is a copolymer of ethylene and methyl acrylate, with a methyl acrylate comonomer content of 24 wt %.

### 2.2. Sample Preparation

PC/PET post-industrial wastes were mechanical treated and grinded to remove the superficial coatings and reduce their dimension, respectively. Scraps of PC/PET were then dried in dehumidifier at 120 °C for 48 hours before dry blending with the compatibilizers and feeding to the extruder. To make the cost of the recycled material comparable to the virgin one, low amounts of the additives were added: the ESAo and MDI contents that were investigated varied from 0 wt % to 5 wt %, while EMAco varied from 0 wt % to 10 wt %. The reactive extrusion experiments were implemented on a co-rotating twin-screw extruder (Collin ZK25, Maitenbeth, Germany), with L/D = 27, D = 25 mm (where L is the screw length and D is the screw diameter) and was operated under vacuum so that all of the resulting light components were forced out continuously. The temperature profile was set to 100–130–200–230–240 °C to avoid overheating and resin degradation [23], and the screw rotation rate was 100 rpm. The blends were cooled into a water bath and cut into pellets using a pelletizer. The pellets were dried at 100 °C for 24 h and then injection molded with a laboratory-scale injection molding machine (Negri Bossi Canbio V55, Milan, Italy) at 260 °C barrel temperature, 60 °C mold temperature, medium injection speed, and 60 bar of injection pressure.

### 2.3. Characterization

The intrinsic viscosity (IV) was measured at 30 °C, based on the ASTM D 4603 standard. The viscosity was performed with an automated viscometer (Lauda iVisc, Delran, NJ, USA), using an Ubbelohde suspended-level viscometer in a phenol/1,1,2,2-tetrachloroethane (60:40 *w*/*w*) solution with a polymer concentration of 0.5 wt %.

Tensile properties were measured with a universal machine (Galdabini SUN2500, Varese, Italy) imposing a uniaxial deformation speed of 50 mm/min for maximum stress and elongation at break, according to UNI ISO 527. The notched Izod impact strength was measured by an impact pendulum (Instron CEAST 9010, Norwood, MA, USA) according to UNI EN ISO 180. The tensile and impact properties of the injection-molded materials were investigated, testing five samples for each kind of the material under study.

Crystallization properties were studied with a differential scanning calorimeter (TA Instruments DSC Q200, New Castle, DE, USA). Samples (about 10 mg taken from the extruded pellets) were cyclically heated and cooled between 30–300 °C under a nitrogen atmosphere, using 10 °C/min as heating and cooling rates; the first heating cycle was used to exclude the effect of the prior thermal history of the extruded samples. The glass transition temperature (T_g_) was determined from the temperature diagrams as the temperature corresponding to the upper inflection point, while the melting temperature (T_m_) and crystallization temperature (T_c_) were determined as corresponding to the maximum of the endothermic curve and the minimum of the exothermic curve, respectively, and crystallinity was calculated by the following equation:

(1) $$X_{cr}\left(\%\right)=\frac{\Delta H_{m}/X_{PET}}{\Delta H_{m}^{*}}\times 100$$

where $\Delta H_{m}^{*}$ = 136 J/g is the melt enthalpy for 100% crystallized PET, $\Delta H_{m}$ is the measured melt enthalpy of the blends (normalized with respect to the additive concentration), and $X_{PET}$ is the PET mass fraction in Xenoy that is equal to 0.21 according to Pratt and Smith [24].

Qualitative chemical analysis and the identification of functional groups in the blend samples were performed using a Fourier transform infrared (FTIR) instrument (Thermo Scientific Nicolet iS50, Waltham, MA, USA), which utilized a diamond crystal as the internal reflection element. The attenuated total reflection (ATR) FTIR spectra were performed in the range of 4000–650 cm^−1^ with solid sample material.

The phase structure was studied using transmission electron microscopy (FEI Tecnai G12, 100 KV with TVIPS Tietz F114 photocamera, Hillsboro, OR, USA). The specimens were prepared using an ultramicrotome with a diamond knife to obtain ultrathin sections of about 100 nm in thickness at room temperature; each sample was taken from the core area of injection-molded bars.

## 3. Results and Discussion

### 3.1. Intrinsic Viscosity (IV)

Table 1 shows the IV values for the blend waste (POST-I) after the extrusion with and without the three different additives; the viscosity of the pure blend was also reported as the target value for the recycling process. The reduction of the IV for the post-industrial blend, with respect to the pure one, is representative of the extended process-induced hydrolytic and thermal decomposition that the polymers suffer during the processing stages. On the other hand, when the additives are added, the effectiveness of the reactive extrusion is proved by the increase in the IV of the samples compared with those of the untreated extrudates. The modification of the molecular structure during reprocessing is reflected in the rheological characteristics of the recycled samples. A significant increase in viscosity due to the addition of MDI and ESAo in POST-I is evident; however, there is only a minor difference in the IV between the POST-I and the blend with different amounts of EMAco. This significant increase in IV suggests that MDI and ESAo actually behave as CEs, and thus enhance the molecular weight of the POST-I blend by rejoining the blend’s broken chains. In particular, the isocyanate groups of MDI and the epoxy groups of ESAo have high reactivity toward the hydroxyl and carboxyl end groups of PET and PC. The behavior shown by the samples with EMAco is different; for this samples the enhancement of the IV is very low, which is in reasonable agreement with the poor reactivity of its functional group.

Concerning the effect of the additive concentration, it was found that the IV of the modified samples increases with the CE content. With the use of MDI, a critical value, above which an increase in the additive concentration prevents a further increase in the IV, was found. This result can be probably ascribed to the high reactivity of the isocyanate group, which can promote secondary reactions that are responsible for the formation of branched structures [25] and/or a cross-linked network [26].

Generally, with the proper amount of the additives, it was possible to restore the IV of POST-I up to values comparable to that of the pure one. Further morphological investigations were needed to understand if the additives act only as chain extenders or also as compatibilizers between PC and PET.

### 3.2 Morphological Properties (Transmission Electron Microscopy)

**Figure 1:**
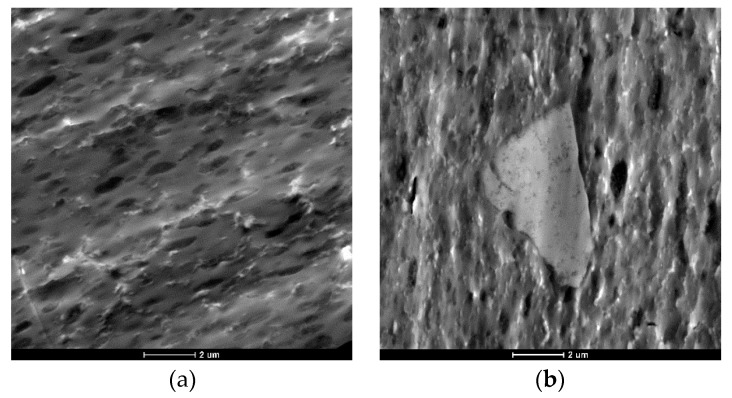
Transmission electron microscopy (TEM) micrographs of: (**a**) pure Xenoy XL1339; (**b**) post-industrial (POST-I) blend.

**Figure 2:**
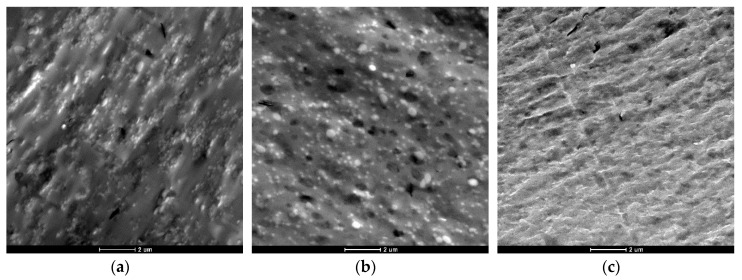
TEM micrographs of: (**a**) POST-I + 5% epoxy styrene-acrylic oligomer (ESAo); (**b**) POST-I + 5% methylene diphenyl diisocyanate (MDI); (**c**) POST-I + 5% ethylene copolymer (EMAco).

To understand the results obtained in the previous section, a transmission electron microscopy (TEM) analysis was employed to compare the morphology of the recycled blends, which can play an important role on their mechanical and physical properties. Figure 1 and Figure 2 show TEM micrographs of treated and untreated samples, respectively.

The surface of the analyzed blend shows a gross phase-separated morphology between the PET droplets and the PC matrix, which is a result of the immiscibility of the blend components and indicates poor interfacial adhesion between the PET and PC phases. The size of PET droplets is in the range of one to two µm. Figure 1b as related to the post-industrial blend displays the presence of some short fibers and other agglomerates, which are probably due to contamination or voluntary introduction during the injection process. 

**Figure 3:**
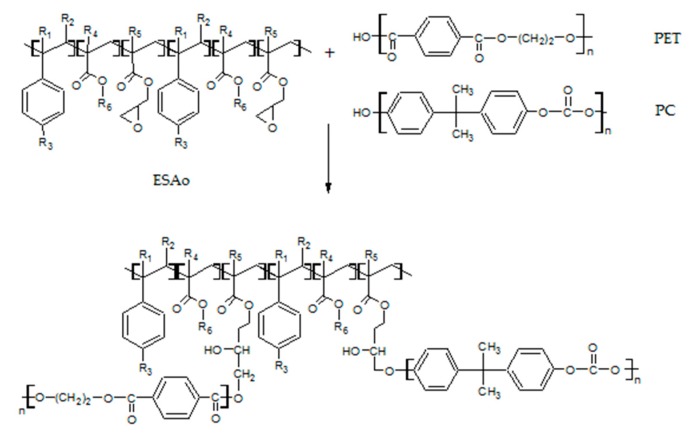
Possible reactions involved during the extrusion of the POST-I blend with ESAo.

**Figure 4:**
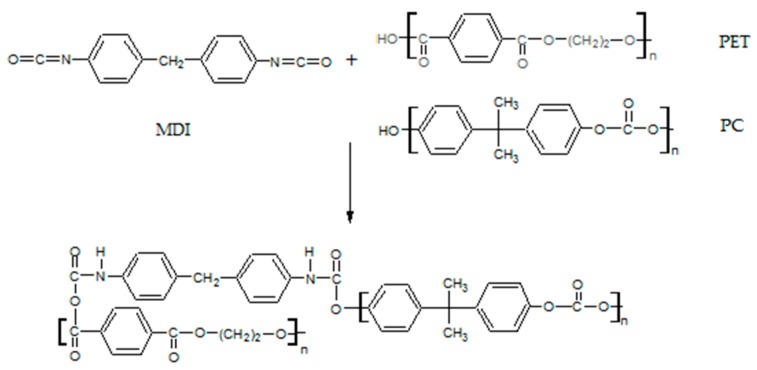
Possible reactions involved during the extrusion of the POST-I blend with MDI.

**Figure 5:**
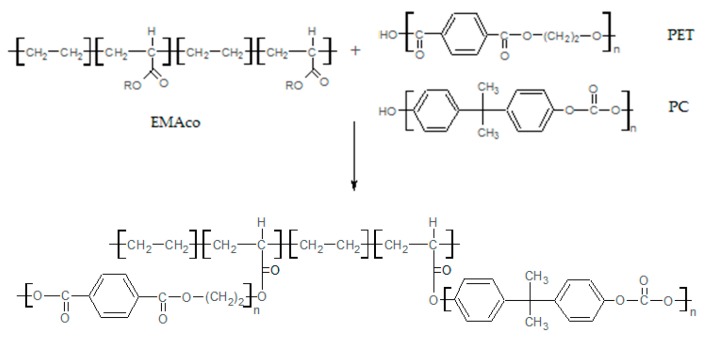
Possible reactions involved during the extrusion of the POST-I blend with EMAco.

It can be seen that the POST-I + 5% ESAo and MDI samples (Figure 2a,b) contain less pull-out cavities and more coalescences of smaller PET aggregates. This difference confirms the compatibilization effect that was induced by the reactions that occurred between the isocyanate and epoxy groups of the additives (MDI and ESAo, respectively) and hydroxyl and carboxyl end groups of the polymers. Instead, the presence of 5% of EMAco produced uniform and homogenous morphology (Figure 2c); this means that the addition of EMAco strongly improved the interfacial adhesion between phases, as well as the miscibility between them. For this reason, the main reactions that can be involved during reactive extrusion are those depicted in Figure 3, Figure 4 and Figure 5. 

As shown in the reaction schemes presented above, the compatibilizers that were studied can react both with PET and PC chains, allowing a better compatibility between the phases. Namely, compatibilizers increase the interactions between the components, and that is manifested in a better phase adhesion [27], as can be seen in the TEM micrographs.

**Figure 6:**
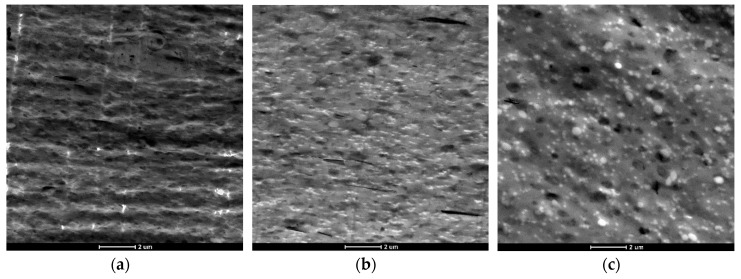
TEM micrographs of POST-I blend with different amount of MDI: (**a**) 2%; (**b**) 3%; (**c**) 5%.

It is interesting to see also how the morphology changes with the use of different amounts of MDI (Figure 6).

When MDI is added in low amounts, there is an increase in the IV, which leads to smaller dimensions of the dispersed phase (Figure 6a,b). This is due to the compatibilization effect induced by the isocyanate groups, which are capable of reacting with both PC and PET terminal groups as shown in Figure 4. However, when it is added in an excessive amount, there is a higher probability to promote dimerization and trimerization reactions between MDI molecules. As result, there is a lower amount of additive available for compatibilization with the possible formation of branched/cross-linked structures. These are responsible for the reduction of the solubility, and therefore of the intrinsic viscosity [26], and for the coarser distribution of the dispersed phase (Figure 6c). 

### 3.3. Thermal Properties

**Figure 7:**
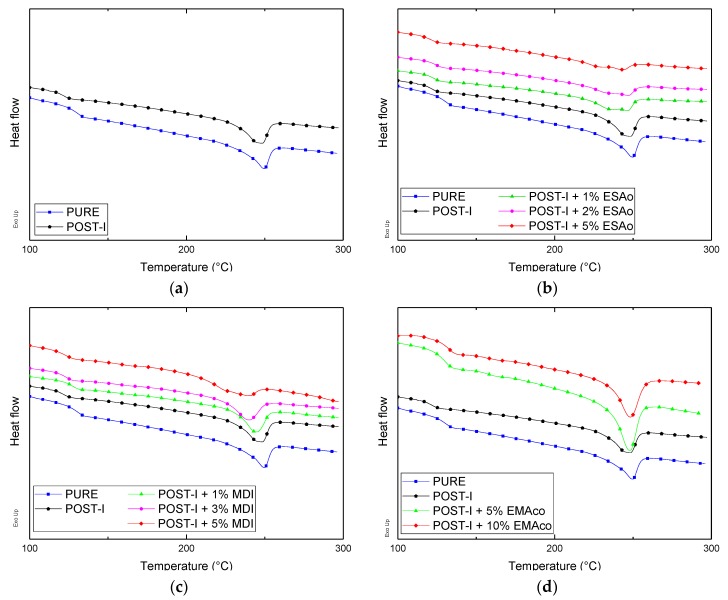
Second heating cycle differential scanning calorimetry (DSC) curves of: (**a**) pure and post-industrial (POST-I) blends without compatibilizers; (**b**) blends with different content of ESAo (1–5 wt %); (**c**) blends with different content of pure MDI (1–5 wt %); (**d**) blends with different content of EMAco (5–10 wt %).

The differential scanning calorimetry thermographs of the blends are shown in Figure 7 and the thermal data are depicted in Table 2. For all of the blends, it was possible to identify only the PC glass transition temperature, whilst the glass transition of the PET was not evident, due to its small concentration in the blend jointly to its partial crystallinity. Indeed, for all of the blends that were analyzed, an endothermic peak was detected at about 250 °C that was associated to PET melting.

The use of MDI and ESAo is reflected on the thermal properties of the extruded blends in the same way. The main effect was that CEs significantly decrease the melting enthalpy and so decrease the crystallinity of PET in ternary blends. The interfacial reaction between the epoxy group of ESAo or the isocyanate group of MDI, and the carboxyl end group of PET and/or the hydroxyl end group of PC, discourages PC/PET chain rearrangement, and consequently cold crystallization. In other words, when ESAo or MDI were introduced in this blending system, the chain-extending reaction among PET and PC would make the molecular weight of the polymers increase; on the other hand, the reactions between PET and PC restricted the movements of PET chains [19,20,26]. As the mobility of the PET chains decreased, PET was more difficult to crystallize than that without CEs, which meant that the crystallization temperature (or rate) decreased. Instead, the effect of EMAco was less pronounced in the thermal properties as it was for the intrinsic viscosity. The crystallization temperature of the blends with this compatibilizer was the highest among all of the samples, suggesting that the presence of EMAco facilitated the crystal nucleation process and resulted in a higher crystallization temperature. The sample with the highest amount of EMAco also showed a slight endothermic peak at 91 °C, which was related to the additive melting temperature.

### 3.4. Fourier Transform Infrared Spectroscopy (FTIR)

**Figure 8:**
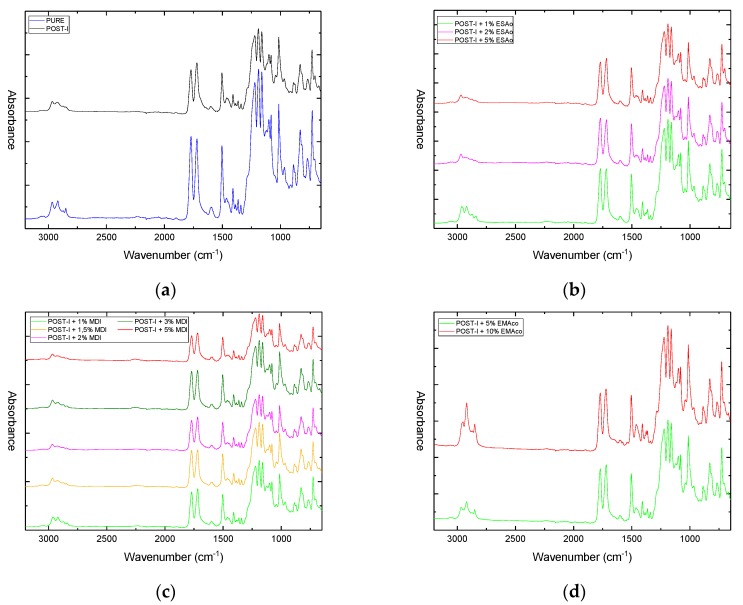
Fourier transform infrared (FTIR) spectra of: (**a**) pure and post-industrial (POST-I) blends without compatibilizers; (**b**) blends with different content of ESAo (1–5 wt %); (**c**) blends with different content of pure MDI (1–5 wt %); (**d**) blends with different content of EMAco (5–10 wt %).

The infrared spectra of the modified and unmodified blends are shown in Figure 8.

A first comparison has been made to highlight the main differences between the virgin Xenoy XL1339 and the POST-I one in order to understand where the degradation phenomenon had occurred and the main findings that were associated to the carbonyl (C=O) stretching region. The peaks at 1770 cm^−1^ and 1719 cm^−1^ correspond to the C=O vibrations of PC and PET, respectively. In the spectra of virgin material, the intensity of the C=O peak that was associated to PC was greater with respect to that of PET, which was in accordance with the higher content of PC in the blend. After industrial treatments, the blend showed an inversion in the intensity of these two peaks, with an additional peak at 1695 cm^−1^ in the form of a shoulder. A possible explanation to this behavior is that PC chains, during both industrial operations and mechanical treatments to remove the coatings, are subjected to degradation toward PC oligomer chains whose carbonyl absorbs at a lower wavenumber, overlapping the PET carbonyl peak. The same occurrence could arise for PET chains, and the carbonyl of oligomeric chains may absorb at 1695 cm^−1^. An alternative explanation offered by Kugo et al. is that the band intensity of the carbonyl group due to PET increased as the molecular weight of PET in a PC/PET blend decreased. In other words, by decreasing the PET molecular weight, there is a surface enrichment of the PET component [28]. In all of the extruded blends, the shoulder at 1695 cm^−1^ disappeared, and the intensity of the peaks, which was related to the two carbonyls, was progressively inverted again, as shown in Table 3. This could also support the previous theory, because the inversion is less marked for the blends that were compatibilized with EMAco, which had shown only a weak molecular weight improvement.

**Figure 9:**
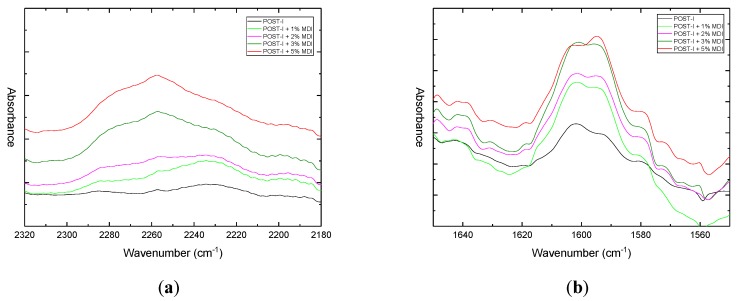
Comparison of FTIR spectra of blends without compatibilizers and with different levels of pure MDI content (1–5 wt %): (**a**) in the region of 2320–2180 cm^−1^; (**b**) in the region of 1650–1550 cm^−1^.

After a reactive extrusion with MDI, the spectra showed distinct differences from the former ones. Two peaks around 2250 cm^−1^ and 1590 cm^−1^ due to isocyanate (–N=C=O) and amide (–NH–CO–) groups were found, both of which increased with the MDI content, as can be seen in Figure 9a and Figure 9b, respectively. The first one was to attributed to an excess of additive, because it is typical of free isocyanate, while the latter may arise from the reaction between isocyanate and the PET acid end groups that occur when an excessive amount of MDI was added [26].

The addition of ESAo did not manifest, in the spectroscopic properties, in a manner differently to how it was already seen. The only exception was for the gradual appearance of a very weak peak at 870 cm^−1^ with the incremental increase of ESAo use, which was linked to the stretching of the epoxy group. The use of EMAco showed only one difference in relation to the other spectra: the occurrence of a peak at 2850 cm^−1^ of the C–H stretching vibration of the acrylic group.

### 3.5. Mechanical Properties

Following the good results obtained with the IV measurements, it was important to also investigate the effect of the compatibilizers on the mechanical properties of the blend waste. The tensile and impact properties are reported in Table 4.

First of all, a deep fall in the mechanical properties of the post-industrial blend compared to the pure one was noted, which was due to the hydrolytic and thermal decomposition that the polymer had been subjected to during the industrial operations. It is probable that other sources for this drastic loss of properties have to also be considered, such as the degradation of some of the compatibilizers that are present in virgin material. The maximum tensile strength remained almost constant with the addition of ESAo and MDI, while EMAco made the post-industrial blend less strong, but slightly more ductile.

Strain at break was subjected to an increase of about 30% thanks to the use of MDI, and no significant differences were noticed upon increasing its amount. The addition of a low amount of ESAo (1 wt %) allowed obtaining the same enhancement of about 30% in the strain at the break value, but increasing the viscosity of the extruded blend caused the material to become stiff. The injection samples obtained with 5 wt % of ESAo showed several delamination defects, which were probably due to the cross-linked structure, and are responsible for the reduction of the mechanical performance. EMAco is more effective in the strain at break improvement.

Similar behavior showed the impact strength. The addition of 1 wt % of ESAo enhanced the impact resistance of the waste blend, but a further increase worsened the property. MDI led to higher Izod values, improving the POST-I value by 90% when it was used at 2 wt %. The lowering in the mechanical properties that occurred beyond a certain level of CE addition might come from phase separation between the additives and matrix in the blends as the CE amount increases [29]. It has to be noted that the improvement in the impact strength thanks to MDI is lower than that described by Tang et al. for the compatibilization of virgin PC and PET [26], which was probably due to the very complex composition of the blend scraps and the possible presence of additive residues that are capable of reacting with the isocyano groups of MDI [30]. EMAco is the compatibilizer that enabled obtaining the best impact strength results with an increase of about 280% with respect to the waste blend value. EMAco is a semicrystalline copolymer, so its crystalline region may act as reinforcement; moreover, the amorphous portions might have behaved similarly to a rubbery phase, contributing to a higher impact resistance [31]. The fact that EMAco did not bring significant changes in viscosity (due to the low reactivity of its functional group) but reduced the brittleness of the samples might be ascribed, according to La Mantia [32], to the different role of this additive. It does not act as a true compatibilizer, but it does reduce the brittleness of at least one of the phases [33]. Another possible reason might be that EMAco acts as a surface-active material that reduces the surface tension between the two polymeric phases during the processing [34], allowing better miscibility, and thus the enhancement of the mechanical performance.

From the mechanical point of view, the materials that are recycled with the following strategy cannot compete with the pure one, but might find their market in areas that do not require the performance of an engineering polymer. However, it might be interesting to evaluate the use of some amounts of those recycled blends within pure Xenoy XL1339 to close the circular economy loop.

## 4. Conclusions

A post-industrial PC/PET blend was recycled by reactive extrusion with different contents of compatibilizers: ESAo, pure MDI, and EMAco. By adding each additive, the compatibility between PET and PC in the post-industrial blend improved, leading to enhanced mechanical properties. EMAco was the compatibilizer that facilitated the best improvements in the mechanical performance of the recycled blend; with the use of only 10 wt % of EMAco, the post-industrial blend tensile strain at break was 30% higher and the Izod value increased by 260%. However, the mechanical behavior of the recycled materials cannot compete with the pure one. Also, the intrinsic viscosity (IV) was improved up to a value that was comparable to that of the pure blend, especially with the use of ESAo and MDI, which act as chain extenders. CEs increase the viscosity of materials, which is explained by the increased molecular weight of the chains as well as by the decelerated crystallization and decreased crystallinity of PET. So, CEs had a dual function in these alloys as both chain extenders and compatibility enhancers. EMAco had a weak impact on the viscosity of the material, but it affects the thermal properties, increasing the crystallization temperature by promoting crystal nucleation at higher temperatures. FTIR analysis of the recycled blends showed the characteristic peak positions of the different organic bonds of the macromolecules. The extra peaks and shifts from the characteristic carbonyl peak positions in the IR spectra indicate the complexity and modification of the molecular structure. Morphological analysis enabled confirming the compatibilization effects induced by the tested additives.

## Figures and Tables

**Table 1:** Intrinsic viscosity (IV) of the materials, before and after recycling methods.

Sample	IV (dL/g)
Xenoy XL1339 pure	0.527 ± 0.022
Xenoy XL1339 POST-I	0.422 ± 0.015
POST-I + 1% ESAo	0.487 ± 0.001
POST-I + 2% ESAo	0.506 ± 0.004
POST-I + 5% ESAo	0.510 ± 0.000
POST-I + 1% MDI	0.455 ± 0.007
POST-I + 1.5% MDI	0.482 ± 0.001
POST-I + 2% MDI	0.497 ± 0.001
POST-I + 3% MDI	0.522 ± 0.000
POST-I + 5% MDI	0.460 ± 0.005
POST-I + 5% EMAco	0.417 ± 0.017
POST-I + 10% EMAco	0.465 ± 0.004

**Table 2:** Thermal characteristic of blends: T_cr_ = crystallization temperature of polyethylene terephthalate (PET); ΔH_cr_ = total heat of crystallization of PET; X_cr_ = degree of crystallinity of PET; T_m_ = melting temperature of PET; ΔH_m_ = total heat of melting of PET; T_g_ PC = polycarbonate glass transition temperature.

Sample	T_cr_ (°C)	ΔH_cr_ (J/g)	X_cr_ (%)	T_m_ (°C)	ΔH_m_ (J/g)	T_g_ PC (°C)
Xenoy XL1339 pure	206	10.5	28.4	250	8.1	132
Xenoy XL1339 POST-I	215	10.5	31.0	247	8.9	122
POST-I + 1% ESAo	204	7.8	27.9	246	8.0	123
POST-I + 2% ESAo	201	6.5	20.5	247	5.9	125
POST-I + 5% ESAo	195	5.2	14.7	243	4.2	121
POST-I + 1% MDI	207	9.8	29.9	244	8.5	128
POST-I + 1.5% MDI	205	8.7	27.9	241	8.0	123
POST-I + 2% MDI	195	8.6	27.1	239	7.8	123
POST-I + 3% MDI	194	7.7	25.1	236	7.2	119
POST-I + 5% MDI	179	2.5	22.4	237	6.4	122
POST-I + 5% EMAco	218	10.5	30.6	248	8.7	131
POST-I + 10% EMAco	218	9.2	27.5	247	7.9	132

**Table 3:** Ratio between PC carbonyl stretching peak (C=O_PC_) and that of PET (C=O_PET_).

Sample	$\frac{{C=O}_{\mathrm{PC}}}{{C=O}_{\mathrm{PET}}}$
Xenoy XL1339 pure	1.05
Xenoy XL1339 POST-I	0.79
POST-I + 1% ESAo	1.02
POST-I + 2% ESAo	1.04
POST-I + 5% ESAo	1.04
POST-I + 1% MDI	0.91
POST-I + 1.5% MDI	1.00
POST-I + 2% MDI	0.98
POST-I + 3% MDI	0.98
POST-I + 5% MDI	0.89
POST-I + 5% EMAco	0.93
POST-I + 10% EMAco	0.94

**Table 4:** Mechanical properties of POST-I blend with different amounts of the compatibilizers that were employed.

Sample	Max Tensile Strength (Mpa)	Strain at Break (%)	Notched Impact Strength (kJ/m^2^)
Xenoy XL1339 pure	51.2 ± 1.8	99.0 ± 17	31 ± 3
Xenoy XL1339 POST-I	53.8 ± 1.2	10.0 ± 1.7	4.7 ± 0.7
POST-I + 1 wt % ESAo	53.1 ± 0.3	12.9 ± 1.6	6.5 ± 1.6
POST-I + 2 wt % ESAo	53.0 ± 0.4	9.3 ± 2.8	4.6 ± 1.7
POST-I + 5 wt % ESAo	44.8 ± 10.0	7.2 ± 4.3	2.9 ± 0.9
POST-I + 1 wt % MDI	53.6 ± 0.4	12.8 ± 1.2	5.5 ± 0.3
POST-I + 1.5 wt % MDI	54.9 ± 1.0	12.3 ± 0.8	6.5 ± 1.9
POST-I + 2 wt % MDI	53.1 ± 0.5	13.8 ± 1.1	9.0 ± 1.2
POST-I + 3 wt % MDI	56.0 ± 0.3	12.8 ± 1.0	7.2 ± 1.5
POST-I + 5 wt % MDI	54.5 ± 0.8	12.3 ± 1.8	7.0 ± 0.7
POST-I + 5 wt % EMAco	48.9 ± 0.5	12.6 ± 1.0	9.4 ± 1.5
POST-I + 10 wt % EMAco	43.4 ± 0.4	15.7 ± 1.7	17.9 ± 1.4
